# Comparative speed of kill after treatment with Simparica^™^(sarolaner) and Advantix^®^(imidacloprid + permethrin) against induced infestations of *Dermacentor reticulatus* on dogs

**DOI:** 10.1186/s13071-016-1377-9

**Published:** 2016-02-24

**Authors:** Csilla Becskei, Thomas Geurden, Heidi Erasmus, Otto Cuppens, Sean P. Mahabir, Robert H. Six

**Affiliations:** Zoetis, Veterinary Medicine Research and Development, Mercuriusstraat 20, Zaventem, B-1930 Belgium; ClinVet International (pty) Ltd, Uitsigweg, Bainsvlei 9338, Bloemfontein, Republic of South Africa; Zoetis, Veterinary Medicine Research and Development, 333 Portage St., Kalamazoo, MI 49007 USA

**Keywords:** *Dermacentor reticulatus*, Sarolaner, Simparica^™^, Imidacloprid + permethrin, Speed of kill, Tick, Dog, Oral, Isoxazoline, Advantix^®^

## Abstract

**Background:**

Ticks are common ectoparasites that infest dogs globally. Acaricides with rapid and sustained speed of kill are critical to control infestations and to reduce the risk of disease transmission. This study evaluated the speed of kill for 5 weeks after a single dose of orally administered Simparica^™^(sarolaner) against induced infestations with *Dermacentor reticulatus* on dogs, compared to Advantix^®^Spot-on solution for dogs (imidacloprid + permethrin).

**Methods:**

Twenty four dogs were randomly allocated to treatment with either a placebo tablet, a sarolaner tablet (at 2 to 4 mg/kg) or with Advantix^®^ as per label instructions. Dogs were treated on Day 0 and tick counts were performed *in situ* at 8 and 12 hours and with removal of the ticks at 24 hours after treatment and subsequent re-infestations on Days 7, 14, 21, 28 and 35. Acaricidal efficacy was determined at each time point relative to live tick counts from the placebo-treated dogs.

**Results:**

Based on arithmetic (geometric) mean tick counts, the efficacy of sarolaner was ≥75.6 % (89.6 %) within 8 hours of treatment and tick counts were significantly lower than placebo and imidacloprid + permethrin-treated dogs (*P <* 0.0001), while imidacloprid + permethrin had no significant reduction (*P ≥* 0.3990) at 8 or 12 hours after treatment. Sarolaner killed all ticks on the dogs within 24 hours after treatment, while imidacloprid + permethrin efficacy was only 48.1 %. After weekly re-infestations sarolaner significantly reduced the tick counts versus placebo within 8 hours on Days 7, 14 and 35 (*P ≤* 0.0239), and at 12 hours and 24 hours (*P ≤* 0.0079) until Day 35.Sarolaner efficacy was ≥95.8 % within 24 hours for 35 days. Significantly more live ticks (*P ≤* 0.0451) were recovered from imidacloprid + permethrin-treated dogs than from sarolaner-treated dogs at 24 hours after infestation on all days. There were no sarolaner-related adverse reactions during the study.

**Conclusions:**

This study demonstrated that Simparica^™^ had a faster and more consistent speed of kill against *D. reticulatus* compared to Advantix^®^. The rapid and consistent efficacy within 24 hours for 5 weeks after a single oral dose of Simparica^™^ provides effective and reliable control of *D. reticulatus* and reduces the risk of transmission of tick-borne diseases.

## Background

*Dermacentor reticulatus,* also known as the ornate dog tick or meadow tick*,* infests dogs throughout Europe. Recently there have been several reports about the potential expansion and increased prevalence of this tick in Europe [1–7]. *D. reticulatus* is also a known vector for *Babesia canis,* an intracellular protozoan parasite that is a threat to dogs as a cause of anemia, thrombocytopenia, and various clinical signs, ranging from mild, nonspecific illness to peracute collapse and death [8, 9]. As awareness of tick-borne diseases has increased, tick control and prevention have taken on a new importance. Until recently, topically administered parasiticides with contact activity have been the only available option for tick control on dogs. These products are generally perceived to have the potential to kill or repel ticks before they bite, reducing the risk of disease transmission.

Simparica^™^(sarolaner) is a novel parasiticide from the isoxazoline class of molecules, and provides a new effective alternative for the control of ticks, including *D. reticulatus* for at least 5 weeks after a single oral dose [10]. As systemically active compounds require the tick to bite to receive a lethal dose of the parasiticide, it is important that these compounds act rapidly and consistently. A single dose of sarolaner provides greater than 90 % efficacy within 24 hours for at least 4 weeks against common ticks worldwide and within 12 hours against *Ixodes ricinus,* the most widely distributed tick species in Europe [11]. While tick efficacy claims are based on evaluation at 48 hours after treatment or re-infestation [12], the speed of acaricidal activity is critical in preventing feeding and thus reducing the risk of disease transmission which occurs after the infected tick is attached and feeding for at least 24 to 48 hours [13, 14]. The present study was conducted to evaluate and compare the speed of kill of a single oral dose of sarolaner with that of imidacloprid + permethrin (Advantix^®^ Spot-on solution for dogs), against an existing infestation and against weekly re-infestations with *D. reticulatus* for a period of 5 weeks after treatment.

## Methods

### Ethical approval

Study procedures were in accordance with the World Association for the Advancement of Veterinary Parasitology (WAAVP) guidelines for evaluating the efficacy of parasiticides for the treatment, prevention and control of flea and tick infestation on dogs and cats [12]. The protocol was reviewed and approved by the Zoetis Ethical Review Board and the local Animal Welfare Committee. Masking of the study was assured through the separation of study functions. All personnel conducting observations or animal care or performing infestations and counts were masked to treatment allocation.

### Animals

Twenty-four (8 male and 16 female) purpose-bred Beagle and mixed breed dogs from 9 months to 6 years of age and weighing from 10.8 to 24.8 kg were used in the study. Each dog had undergone an adequate wash-out period to ensure that no residual ectoparasiticide efficacy remained from any previous administration. Dogs were individually housed and were acclimatized to these conditions for at least 8 days prior to treatment. Dogs were fed an appropriate maintenance ration of a commercial canine diet for the duration of the study. Water was available *ad libitum*. All dogs were given a physical examination to ensure that they were in good health at enrollment and were suitable for inclusion in the study. General health observations were performed twice daily throughout the study.

### Design

The study followed a randomized complete block design. Dogs were ranked according to decreasing tick counts into blocks of three animals, and within each block a dog was randomly allocated to one of three treatment groups. There were eight dogs per treatment group. The average hair length in each group was similar.

### Treatment

On Day 0, dogs received either a placebo tablet, an appropriate Simparica^™^ chewable tablet (sarolaner at 2 to 4 mg/kg) or a topical application of Advantix^®^ Spot-on solution for dogs (imidacloprid + permethrin at 10 to 25 mg/kg imidacloprid and 50 to125 mg/kg permethrin). The Day -2 bodyweights were used to calculate the appropriate dosage to be administered. The tablet(s) were administered by hand pilling to ensure accurate and complete dosing. Each dog was observed for several minutes after dosing for evidence that the dose was swallowed, and for potential adverse events associated with treatment administration. Dogs were observed approximately two hours after dosing for evidence of emesis. The imidacloprid + permethrin treatment was applied topically following the label directions.

### Tick infestation and assessment

The *D. reticulatus* strain used for infestation was established in 2007 in Ireland. The colony was genetically enriched by the addition of wild-caught ticks from the Netherlands in 2009 and 2012. Tick infestations were performed on Days -7,-2, 7, 14, 21, 28 and 35. Prior to each infestation, the dog was sedated to enhance tick attachment, and 50 (±5) viable unfed adult *D. reticulatus* (1:1 male:female) was directly applied to each animal. Each dog was examined to remove and count live ticks at 48 hours after the infestation on Day -7, to confirm host suitability and counts were used for allocation to treatments. On Days 0, 7, 14, 21, 28 and 35, the dogs were examined 8 and 12 (±0.5) hours after treatment or each subsequent weekly re-infestation, and live ticks were counted *in situ*; the dogs were systematically examined so that the entire body surface was carefully examined once. At 24 (±0.5) hours after treatment and each subsequent weekly re-infestation, the dogs were examined and then thoroughly combed to count and remove ticks. Each dog was examined for at least 10 minutes. If ticks were encountered in the last minute, combing was continued in one minute increments until no ticks were encountered.

### Statistical analysis

The individual dog was the experimental unit. Data for post-treatment live (free plus attached) tick counts were summarized with arithmetic (AM) and geometric (GM) means by treatment group and time-point. Tick counts were transformed by the log_e_ (count + 1) transformation prior to analysis in order to stabilize the variance and normalize the data. Using the PROC MIXED procedure (SAS 9.2, Cary NC), transformed counts were analyzed using a mixed linear model for repeated measures. The fixed effects were treatment, time-point and the treatment by time-point interaction. Random effects included room, block within room, block by treatment interaction within room, and error. Testing was two-sided at the significance level α = 0.05.

The assessment of acaricidal efficacy was based on the percent reduction in the arithmetic and geometric mean live tick counts relative to placebo, as suggested by the most recent WAAVP guidelines [12], and was calculated using Abbott’s formula:

$$ \%\ \mathrm{reduction}=100 \times \frac{\mathrm{mean}\ \mathrm{count}\ \left(\mathrm{placebo}\right)\hbox{--}\ \mathrm{mean}\ \mathrm{count}\ \left(\mathrm{treated}\right)}{\mathrm{mean}\ \mathrm{count}\ \left(\mathrm{placebo}\right)} $$

## Results

**Fig. 1:**
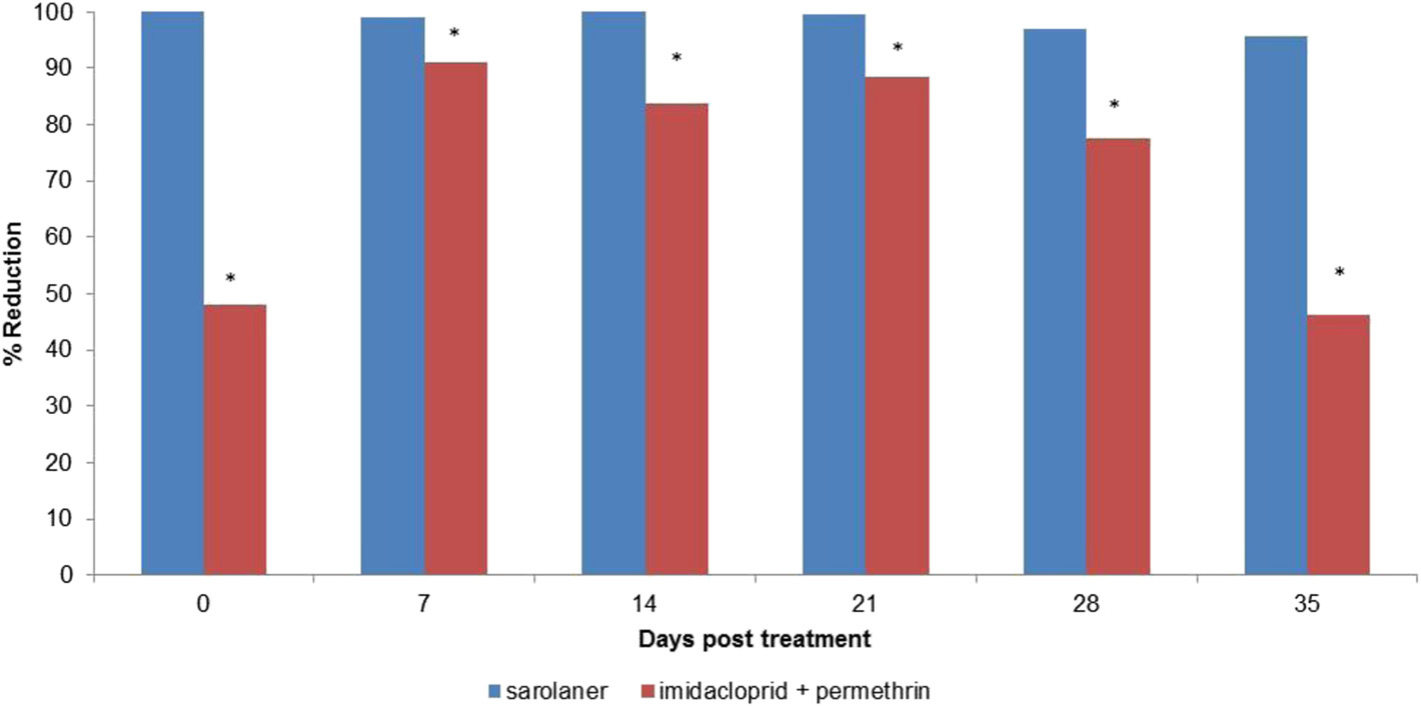
Percent efficacy based on arithmetic mean *Dermacentor reticulatus* counts relative to placebo at 24 hours after treatment and weekly post-treatment re-infestation for dogs treated with either a single oral dose of sarolaner or a single topical application of imidacloprid plus permethrin on Day 0

The results of the tick counts at each time-point are provided in Tables 1, 2 and 3, and in Fig. 1. Placebo-treated dogs maintained adequate tick infestations throughout the study. AM (GM) live tick counts for the placebo-treated dogs at the 8-hour tick count ranged from 23.5 (21.7) to 35.8 (34.5); at the 12-hour count from 20.5 (18.3) to 33.6 (32.3); and at the 24-hour count from 25.0 (23.0) to 30.1 (28.7).

**Table 1:** The Range, Arithmetic (AM) and Geometric (GM) Mean live *Dermacentor reticulatus* counts, and efficacy relative to placebo at 8 hours after treatment and re-infestations for dogs treated with either a single oral dose of sarolaner or a single topical application of imidacloprid+permethrin on Day 0

Treatment		Day of treatment or re-infestation					
		0	7	14	21	28	35
Placebo	Range	23 to 43	12 to 34	26 to 43	13 to 40	21 to 39	20 to 43
	AM	33.8	23.5	35.8	28.4	32.4	35.4
	GM^c^	32.4^a^	21.7^a^	34.5^a^	26.4^a^	31.2^a^	33.7^a^
Sarolaner	Range	0 to 31	6 to 21	8 to 22	12 to 27	13 to 30	12 to 33
	AM	8.3	13.1	12.5	19.9	22.1	21.1
	AM Efficacy (%)	75.6	44.1	65.0	30.0	31.7	40.3
	GM^c^	3.4^b^	12.1^b^	11.6^b^	18.6^a^	20.6^a^	19.8^b^
	GM Efficacy (%)	89.6	44.1	66.4	29.6	33.9	41.2
	*P*-value vs. placebo	<0.0001	0.0157	<0.0001	0.1349	0.0766	0.0239
Imidacloprid+permethrin	Range	6 to 45	0 to 33	2 to 18	0 to 18	5 to 20	7 to 42
	AM	31.9	9.9	8.9	6.9	14.3	19.5
	AM Efficacy (%)	5.6	58.0	75.2	75.8	56.0	44.9
	GM^c^	27.6^a^	4.2^c^	7.2^b^	4.7^b^	12.8^b^	16.8^b^
	GM Efficacy (%)	14.6	80.6	79.0	82.0	58.9	50.2
	*P*-value vs. placebo	0.6216	<0.0001	<0.0001	<0.0001	0.0074	0.0331
	P-value vs. sarolaner	<0.0001	0.0077	0.2152	0.0005	0.1917	0.6461

^c^ Geometric means within columns with the same superscript are not significantly different (*P* >0.05)

**Table 2:** The Range, Arithmetic (AM) and Geometric (GM) Mean live *Dermacentor reticulatus* counts, and efficacy relative to placebo at 12 hours after treatment and re-infestations for dogs treated with either a single oral dose of sarolaner or a single topical application of imidacloprid+permethrin on Day 0

Treatment		Day of treatment or re-infestation					
		0	7	14	21	28	35
Placebo	Range	23 to 41	6 to 35	23 to 41	12 to 37	19 to 34	12 to 43
	AM	33.6	20.5	31.3	27.0	28.5	33.3
	GM^c^	32.3^a^	18.3^a^	30.1^a^	25.2^a^	27.3^a^	30.6^a^
Sarolaner	Range	0 to 7	3 to 15	6 to 13	1 to 24	9 to 24	10 to 28
	AM	1.4	8.6	8.6	11.0	15.6	16.9
	AM Efficacy (%)	95.9	57.9	72.4	59.3	45.2	49.2
	GM^c^	0.6^b^	7.7^b^	8.2^b^	8.4^b^	14.5^b^	15.7^b^
	GM Efficacy (%)	98.0	58.0	72.7	66.8	47.0	48.7
	*P*-value vs. placebo	<0.0001	0.0005	<0.0001	<0.0001	0.0079	0.0051
Imidacloprid+permethrin	Range	6 to 41	0 to 29	0 to 16	0 to 10	3 to 16	7 to 36
	AM	28.1	7.5	6.3	4.4	8.4	17.5
	AM Efficacy (%)	16.4	63.4	80.0	83.8	70.6	47.4
	GM^c^	24.6^a^	3.5^b^	4.2^b^	3.3^c^	7.2^b^	14.7^b^
	GM Efficacy (%)	23.7	80.8	85.9	87.0	73.8	51.9
	*P*-value vs. placebo	0.3990	<0.0001	<0.0001	<0.0001	0.0001	0.0259
	*P*-value vs. sarolaner	<0.0001	0.0578	0.1025	0.0231	0.0630	0.8596

^c^ Geometric means within columns with the same superscript are not significantly different (*P* >0.05)

**Table 3:** The Range, Arithmetic (AM) and Geometric (GM) Mean live *Dermacentor reticulatus* counts, and efficacy relative to placebo at 24 hours after treatment and re-infestations for dogs treated with either a single oral dose of sarolaner or a single topical application of imidacloprid+permethrin on Day 0

Treatment		Day of treatment or re-infestation					
		0	7	14	21	28	35
Placebo	Range	21 to 40	10 to 37	15 to 37	15 to 38	18 to 33	7 to 41
	AM	30.1	25.0	28.4	28.1	28.0	27.0
	GM^d^	28.7^a^	23.0^a^	26.6^a^	26.7^a^	26.9^a^	24.0^a^
Sarolaner	Range	0 to 0	0 to 1	0 to 0	0 to 1	0 to 3	0 to 5
	AM	0.0	0.3	0.0	0.1	0.9	1.1
	AM Efficacy (%)	100.0	99.0	100.0	99.6	96.9	95.8
	GM^d^	0.0^c^	0.2^c^	0.0^c^	0.1^c^	0.5^c^	0.6^c^
	GM Efficacy (%)	100	99.3	100	99.8	98.0	97.6
	*P*-value vs. placebo	<0.0001	<0.0001	<0.0001	<0.0001	<0.0001	<0.0001
Imidacloprid+permethrin	Range	7 to 33	0 to 8	0 to 18	0 to 11	0 to 17	1 to 32
	AM	15.6	2.3	4.6	3.3	6.3	14.5
	AM Efficacy (%)	48.1	91.0	83.7	88.4	77.7	46.3
	GM^d^	13.9^b^	1.3^b^	2.3^b^	1.9^b^	4.1^b^	10.4^b^
	GM Efficacy (%)	51.5	94.3	91.3	93.0	84.9	56.8
	*P*-value vs. placebo	0.0282	<0.0001	<0.0001	<0.0001	<0.0001	0.0124
	*P*-value vs. sarolaner	<0.0001	0.0451	0.0005	0.0045	0.0006	<0.0001

^d^ Geometric means within columns with the same superscript are not significantly different (*P* >0.05)

Eight hours after treatment the reduction in AM (GM) tick counts was 75.6 % (89.6 %) for sarolaner and 5.6 % (14.6 %) for imidacloprid + permethrin. Geometric mean live tick counts were significantly lower (*P <* 0.0001) than placebo for sarolaner but not forimidacloprid + permethrin (*P =* 0.6216).Eight hours after the subsequent weekly re-infestations the reduction in AM (GM) tick counts for sarolaner ranged from 30.0 % (29.6 %) to 65.0 % (66.4 %). GM live tick counts for sarolaner were significantly lower (*P ≤* 0.0239) than placebo, except on Days 21 and 28. Forimidacloprid + permethrin, the reduction in AM (GM) mean tick counts ranged from 44.9 % (50.2 %) to 75.8 % (82.0 %), and were significantly lower (*P ≤* 0.0331) than placebo on all days. GM live tick counts for the two products were significantly different from each other on Days 0, 7 and 21 (*P ≤* 0.0077).

Twelve hours after treatment the reduction in AM (GM) tick counts was 95.9 % (98.0 %) for sarolaner and 16.4 % (23.7 %) for imidacloprid + permethrin. GM live tick counts for sarolaner were significantly lower (*P <* 0.0001) than for placebo, but were not for imidacloprid + permethrin (*P =* 0.3990) versus placebo. On the sarolaner-treated dogs only up to 7 live ticks were found, while on the imidacloprid + permethrin treated dogs up to 41 live ticks were recovered. Twelve hours after weekly re-infestation the reduction in AM (GM) tick counts for sarolaner ranged from 45.2 % (47.0 %) to 72.4 % (72.7 %) (*P ≤* 0.0079 on all days vs placebo). Forimidacloprid + permethrin, the reduction in AM (GM) mean tick counts ranged from 47.4 % (51.9 %) to 83.8 % (87.0 %) (*P ≤* 0.0259 on all days vs. placebo). GM live tick counts for sarolaner were significantly lower than those for imidacloprid + permethrinon Days 0 and 21 (*P ≤* 0.0231).

Twenty four hours after treatment no live ticks were found on any sarolaner-treated dog (100 % efficacy), while up to 33 live ticks were recovered from imidacloprid + permethrin treated dogs, representing 48.1 % (51.5 %) efficacy. GM live tick counts were significantly lower than placebo for sarolaner (*P <* 0.0001) and imidacloprid + permethrin (*P ≤* 0.0282). Twenty four hours after weekly re-infestation, the reduction in AM (GM) tick counts for sarolaner was above 95.8 % (97.6 %) until Day 35 (*P <* 0.0001 vs. placebo on all days). Forimidacloprid + permethrin, the percentage reduction in AM (GM) tick counts ranged from 91.0 % (94.3 %), to 46.3 % (56.8 %) (*P ≤* 0.0124 vs. placebo on all days). Geometric mean live tick counts for sarolaner were significantly lower than those for imidacloprid + permethrin on all days (*P ≤* 0.0451).There were no sarolaner-related adverse reactions during the study. One imidacloprid + permethrin-treated dog developed erythema on Day 0 at the site of treatment application.

## Discussion

A rapid speed of kill prevents the direct adverse effects of tick attachment and feeding, and reduces the risk for transmission of tick-borne pathogens. In this study, a single dose of sarolaner significantly reduced *D. reticulatus* tick counts within 8 hours after treatment, and within 12 hours after re-infestation for 35 days. Efficacy above 90 % was achieved within 24 hours and maintained for 35 days after treatment. The persistent and rapid speed of kill against *D. reticulatus* for 5 weeks is consistent with the rapid efficacy of sarolaner within 24 hours against *Ixodes ricinus*, *Ixodes scapularis* and *Amblyomma maculatum* for at least 4 weeks [11].

The efficacy of imidacloprid + permethrin within 48 hours of treatment against *D. reticulatus* in dogs has previously been described, with adequate efficacy in the first two weeks after treatment and a decline in efficacy from the third week onwards [15, 16]. Additionally, the efficacy of imidacloprid + permethrin at 24 hours post-treatment or re-infestation was below 90 % efficacy throughout the entire month in another study [17]. Similarly, in the present study, based on arithmetic mean the efficacy of imidacloprid + permethrin at 24 hours was below 90 % at all time-points except on Day 7. As a result, significantly (*P ≤* 0.0451) more ticks were found at 24 hours on imidacloprid + permethrin-treated dogs compared to sarolaner-treated dogs throughout the study. The fact that imidacloprid + permethrin had no significant effect within 12 hours after treatment and <50 % efficacy at 24 hours, as well as the lower efficacy throughout the month at all time-points except at 24 hours on Day 7, are evidence of a slow speed of kill for imidacloprid + permethrin.

The ornate dog tick is a well-known vector of *B. canis*. Transmission of babesiosis is considered to occur within 24 to 72 hours of tick attachment [13, 14]. As dogs can be exposed to infected ticks throughout the treatment interval, both persistent efficacy and rapid speed of kill are required to decrease the risk of babesiosis. Under field conditions imidacloprid + permethrin has demonstrated a 94.4 % reduction in the incidence of infection with *Babesia spp* in dogs [18]. Therefore the faster and more persistent killing effect of sarolaner against *D. reticulatus* for at least 5 weeks in the present study highlights the potential of sarolaner to reduce the transmission of babesiosis to dogs.

## Conclusions

This study confirmed the rapid and consistent acaricidal efficacy of sarolaner against *D. reticulatus* after a single oral administration and demonstrated that ticks were killed rapidly with >90 % efficacy within 24 hours for at least 5 weeks. Efficacy of Simparica^™^ was higher at 24 hours compared to Advantix^®^ throughout the study.
